# Treatment of Refractory/High-Risk Pregnancies With Antiphospholipid Syndrome: A Systematic Review of the Literature

**DOI:** 10.3389/fphar.2022.849692

**Published:** 2022-05-19

**Authors:** Ariela Hoxha, Daniela Tormene, Elena Campello, Paolo Simioni

**Affiliations:** ^1^ Internal Medicine Unit, Department of Medicine, San Bortolo Hospital, Vicenza, Italy; ^2^ General Internal Medicine and Thrombotic and Hemorrhagic Unit, University of Padua, Padua, Italy

**Keywords:** obstetric antiphospholipid syndrome, pregnancy, antiphospholipid syndrome, therapy, antiphospholipid antibodies

## Abstract

Different treatment protocols have been employed to manage heparin/low-dose aspirin refractory or high-risk pregnancies in antiphospholipid antibody syndrome (APS) pregnancies. A systematic review of the literature on additional treatments used in refractory and/or high-risk APS pregnancies was conducted. Records from February 2006 to October 2021 were retrieved from PubMed, Web of Science, Cochrane, and the www.clinicaltrials.gov platform. Twenty-one studies met our eligibility criteria. Live birth rate is this study’s primary endpoint, while pregnancy complications and adverse events are secondary endpoints. A total of 434 pregnancies, 162 (37.3%) refractory and 272 (62.7%) high-risk/refractory pregnancies, were included. Both IVIG <2 gr/kg/monthly/HCQ/LDS and PEX/IA ± LDS led to 100% viable infants in refractory APS. Furthermore, HCQ 200–400 mg showed a higher live birth rate than HCQ + LDS (88.6% *vs*. 82.7%). Following treatment protocol with HCQ 200–400 mg and IVIG <2 gr/kg/monthly/HCQ/LDS, pregnancy complications rates of 16.7 and 83.3% were registered, respectively. Pravastatin 20 mg, IA weekly + IVIG 2 gr/monthly, and PEX weekly + IVIg 2 gr/kg/monthly showed higher live birth rates in high-risk APS pregnancies of 100, 100 and 92%, respectively, whereas the lower severe pregnancy complications were reported in pregnancies treated with PEX weekly + IVIg 2 gr/kg/monthly (11.1%). One (0.6%) case of dermatitis during treatment with HCQ was observed. The results of this study showed that HCQ 200–400 mg and PEX weekly + IVIG 2 gr/kg/monthly achieved a higher live birth rate in refractory APS and high-risk/refractory APS, respectively. The results presented provide clinicians with up-to-date knowledge in the management of APS pregnancies according to risk stratification.

## Introduction

Pregnancy morbidity is one of the clinical hallmarks of antiphospholipid syndrome (APS). Obstetric APS is defined as a) one or more unexplained deaths of morphologically normal fetuses at or beyond the 10th week of gestations and/or b) one or more premature births of morphologically normal neonates before the 34th week of gestation because of preeclampsia or severe eclampsia or signs of placental insufficiency and/or c) three or more unexplained consecutive spontaneous abortions before the 10th week of gestations with maternal anatomic or hormonal abnormalities and paternal and maternal chromosomal causes excluded (Miyakis et al., 2006). Obstetric APS morbidity, one of the few treatable causes of recurrent loss, is a challenging issue for clinicians and is a major health burden for women of childbearing age. (Pengo et al., 2016; Schreiber K and Hunt BJ. 2016). Currently, the European League Against Rheumatism (EULAR) guidelines for the management of APS recommends heparin with low-dose aspirin (LDA) as the standard of care (SoC) to prevent pregnancy loss (Tektonidou et al., 2019), even though the evidence is of low certainty (Hamulyák et al., 2021). Following this treatment up to 70–80% of viable live infants could be achieved (Bramham et al., 2010; Bouvier et al., 2014). In the 20–30% of failed pregnancies, a series of risk factors associated with unfavorable pregnancy outcomes have been described (Sailer et al., 2006; Ruffatti et al., 2011; Lockshin et al., 2012; Matsuki et al., 2015; Latino et al., 2017; Saccone et al., 2017; Walter et al., 2021). Recently, both inflammatory and non-inflammatory pathogenetic mechanisms are described in APS placenta studies (Tong et al., 2015; Vial and Chamley 2015), reflecting the different characteristics of obstetric features. An inadequate invasion of the maternal spiral arteries by the extravillous cytotrophoblast and severe inflammation of the placenta, leading to early miscarriages, might explain early pregnancy loss in APS patients; on the other hand, an impaired transformation of the maternal spiral arteries together with complement and coagulation cascade activation might be responsible for late pregnancy loss and preeclampsia. Thus, it is crucial to stratify the risk of pregnancy failure in APS women in order to personalize the treatment, leading to a higher live birth rate and fewer pregnancy complications. A series of additional therapies have been employed to improve pregnancy outcomes (El-Haieg DO et al., 2007; Bramham et al., 2011; Mekinian et al., 2015; Lefkou et al., 2016; Ruffatti et al., 2018; Alijotas-Reig et al., 2019; Hoxha et al., 2020); however, which is the correct strategy is still a matter of debate (Giacomelli et al., 2021; Ruffatti et al., 2021). We conducted this systematic review of the literature to evaluate the most efficacious additional treatments, according to risk stratification in primary APS patients.

## Materials and Methods

This systematic review of the literature was performed in accordance with the general principles of the Preferred Reporting Items for Systematic Reviews and Meta-Analysis (PRISMA). The PRISMA checklist is provided in online Supplementary Table S1 (Page et al., 2021).

### Search Strategy

We performed a detailed search in the following databases for original articles: PubMed, Web of Science, the Cochrane database, and www.clinicaltrials.gov. The period examined was February 2006-October 2021. We adopted a PICO strategy of pre-defined essential elements [“Population = women with refractory or high-risk primary obstetric APS,” “Intervention = additional treatments to conventional therapy,” “Comparison = with a control group on placebo or on different therapy,” “Outcomes = outcome of pregnancy (live birth, pregnancy loss, maternal complications, fetal complications, and side effects)”] for study inclusion in order to describe the most effective and safe additional treatment inducing a favorable pregnancy outcome in women with refractory or high-risk obstetric APS.

The search strategy combined free text search, exploded medical subject heading (MESH/EMTREE) terms, and all synonyms of the following MESH terms to identify relevant published articles: “antiphospholipid syndrome,” “refractory antiphospholipid syndrome,” and “pregnancy” in combination with “additional treatments,” “hydroxychloroquine,” “low-dose steroids,” “intravenous immunoglobulins,” “plasma exchange,” “rituximab,” “eculizumab,” “certolizumab,” “adalimumab,” “etanercept,” and “statins”. The computerized search was completed with a manual search of pertinent reference lists from the relevant articles retrieved. Articles written in languages other than English were excluded.

Two main investigators (HA and CE) reviewed independently the literature. In case of discrepancy, a third external reviewer (TD) made the final decision.

### Study Identification and Data Extraction

Selection criteria were determined before data collection. Studies were considered eligible if they met the following criteria: 1) primary APS patients defined following the Sydney Consensus Statement (Miyakis et al., 2006); 2) refractory and/or high-risk pregnancies treated with additional treatment protocol in association with the SoC; 3) either observational or interventional studies reporting on the treatment of refractory and/or high-risk pregnancies treated with additional treatment protocols.

The women without APS laboratory and clinical risk factors, who experienced pregnancy failure while being treated with heparin/LDA, were defined as “refractory obstetric APS,” whereas APS patients with one or more laboratory and/or clinical risk factors who may or may not have experienced adverse pregnancy outcomes despite treatment with heparin/LDA were defined as “high-risk and/or refractory obstetric APS”. In accordance with the literature (Sailer et al., 2006; Ruffatti et al., 2011; Lockshin et al., 2012; Matsuki et al., 2015; Latino et al., 2017; Saccone et al., 2017; Walter et al., 2021) laboratory risk factors were considered: persistent LA positivity alone or any combination of LA with IgG/IgM aCL antibodies or IgG/IgM anti-β2 glycoprotein I antibodies and triple aPL positivity (LA + IgG/IgM aCL antibodies + IgG/IgM anti-β2GPI antibodies). As highlighted in the literature (Ruffatti et al., 2011; Walter et al., 2021), thrombosis, systemic lupus erythematosus, and previous severe pregnancy complications were considered clinical risk factors. Severe pregnancy complications referred to one or more of the following: eclampsia, preeclampsia (new-onset hypertension ≥140/90 mm Hg and proteinuria ≥300 mg per 24-h urine collection protein), placental insufficiency (abnormal Doppler flow velocimetry waveform analysis of uterine arteries), hemolysis, elevated liver enzymes, low platelet count (HELLP) syndrome, and intrauterine growth retardation (IUGR) defined as postnatal birth weight less than 10th percentile for gestational age.

Reviews, editorials, articles on pregnancies in patients affected with systemic lupus erythematosus, articles considering the aforementioned therapies as first-line treatment to prevent pregnancy morbidity in APS, and articles with poor or non-documented follow-up were excluded.

Full-text articles were screened and selected, analyzing titles and abstracts. After the screening phase, the selected abstracts and the full text of these studies were evaluated to determine eligibility. Articles retrieved by the literature search but reporting insufficient data, according to the selected PICO strategy, were excluded. The online search was limited to randomized clinical trials, case-control, cohort, and case-series studies; however, given the rarity of the condition, case reports were also selected.

The full text of the selected studies was retrieved, and data were extracted from an electronic database, summarized, analyzed, and discussed. The study’s primary endpoint was live birth rate; pregnancy complications and drug adverse events were secondary endpoints.

### Quality Assessment

The quality assessment of the case-control and cohort studies was evaluated using the Newcastle–Ottawa scale (Wells et al., 2019), respectively, for case-control and cohort studies. The studies that scored at least five stars were considered to have moderate-high methodological quality. Johanna-Briggs Institute critical appraisal tools (Johanna-Briggs Institute 2017) were used to assess the quality of case reports and case series. The levels of evidence, suggested by Oxford University, were followed to identify the hierarchy of study types (http://www.cebm.net/oxford-centre-evidencebased-me dicine-levels-evidence-february-2022/).

### Statistical Analysis

Statistical analysis was performed by GraphPad Prism version 5.00 (San Diego, California, United States). Data are shown as numbers (percentages).

## Results

**FIGURE 1:**
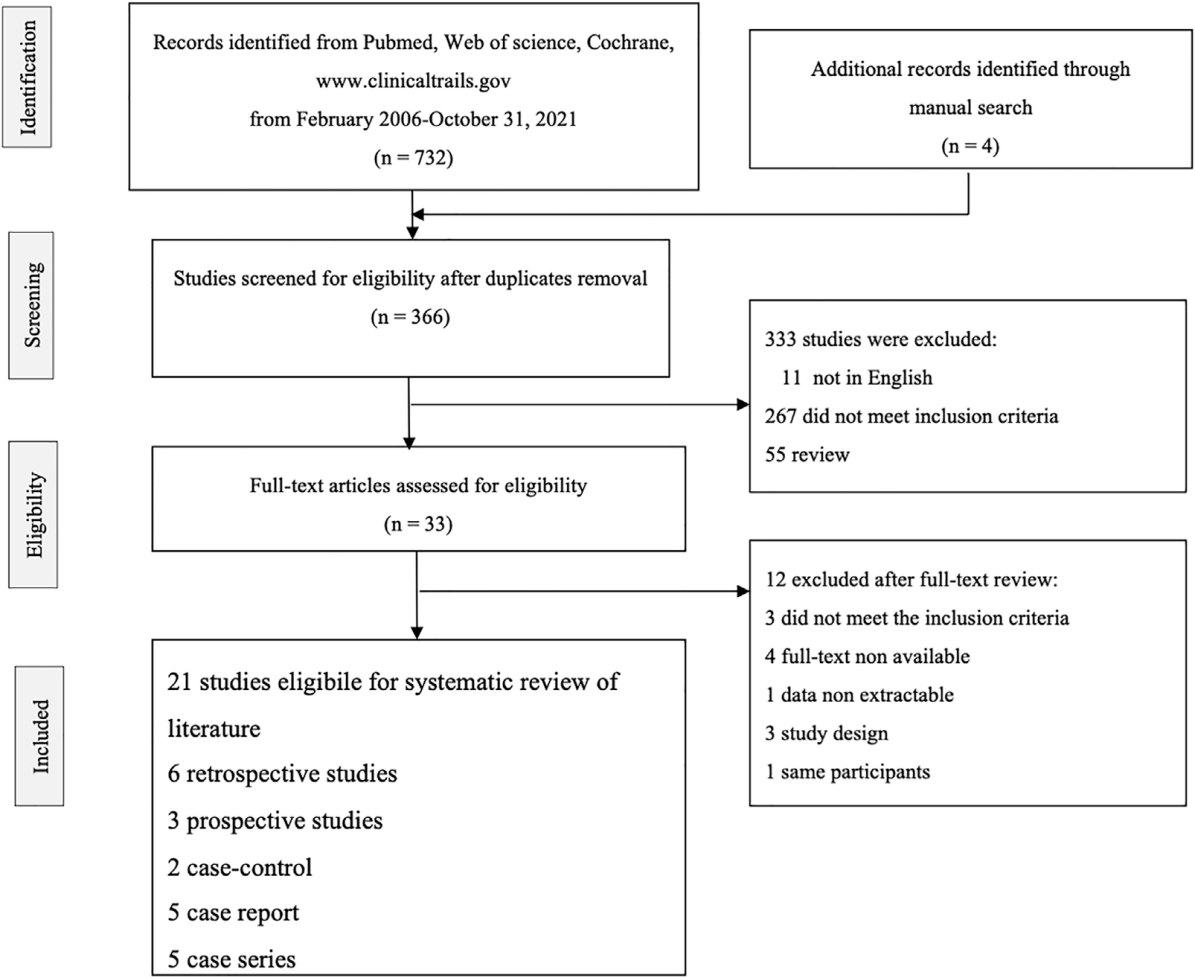
Flow diagram of study selection.

As illustrated in the flow diagram (Figure 1), 366 records of abstracts were retrieved by the electronic database search and manual search of references after the elimination of duplicates between databases. Among them, 333 were excluded after title and abstract screening because they were not in English, did not meet the inclusion criteria, or were not observational studies or clinical trials. Thirty-three articles underwent a further full-text review for eligibility. Twelve studies were excluded:

Three studies (Abisror et al., 2014; Gan et al., 2016; Gustavsen et al., 2017) did not meet the inclusion criteria, four studies (Nagata et al., 2020; Gerde et al., 2021; Khizroeva et al., 2021; Kupferminc et al., 2021) because the full-text was not available, one study (Sciascia et al., 2016) because the data were not extractable, three studies (Schreiber et al., 2017; Belizna et al., 2018; Pasquier et al., 2019) because of the study design of ongoing clinical trials, and one study (Hoxha et al., 2020) as it considered the same participants.

In the end, 21 studies were eligible for the systematic review of the literature:

Six retrospective studies (Ruffatti et al., 2014; Mekinian et al., 2015; Ruffatti et al., 2015; Mekinian et al., 2017; Ruffatti et al., 2018; Colpo et al., 2019), three prospective studies (Bramham et al., 2011; Ruffatti et al., 2016; Alijotas-Reig et al., 2019), two case-control studies (Lefkou et al., 2016; Lefkou et al., 2020), five case reports (Stojanovic et al., 2007; Lefkou et al., 2014; De Carolis et al., 2015; Mayer-Pickel et al., 2015; Rovere-Querini et al., 2018) and five case series (El Haieg et al., 2007; Ruffatti et al., 2007; Watanabe et al., 2014; Mayer-Pickel et al., 2017; Urban et al., 2020).

In total, 434 refractory and high-risk/refractory pregnancies were included in the study. Of these, 162 (37.3%) were refractory, and 272 (62.7%) were high-risk/refractory APS pregnancies.

### Quality Score

The six retrospective, three prospective, and two case-control studies were assessed with the Newcastle–Ottawa scale (Wells et al., 2019) which assigned a high-quality rating to seven of the studies (Bramham et al., 2011; Ruffatti et al., 2014; Lefkou et al., 2016; Ruffatti et al., 2016; Ruffatti et al., 2018; Colpo et al., 2020; Lefkou et al., 2020) and a moderate quality rating to four of the studies (Mekinian et al., 2015; Ruffatti et al., 2015; Mekinian et al., 2017; Alijotas-Reig et al., 2019). The five case reports and five case series were evaluated with Johanna–Briggs critical appraisal tools (Johanna-Briggs Institute 2017) and deemed eligible.

### Additional Treatment to Prevent Pregnancy Failure in Refractory APS Pregnancies

The studies investigating the effects of additional treatments in the refractory APS pregnancies are reported in Table 1.

**TABLE 1:** Studies investigating the effects of additional therapies in refractory antiphospholipid syndrome (APS) pregnancies.

Author, year	Study design	Additional treatment protocol	Number of pregnancies	aPL profile	Pregnancy morbidity, (n)	Thrombosis, (n)	Live birth, n (%)	Pregnancy complications, n (%)	Adverse events, n (%)	Level of evidence
El Haieg et al. (2007)	case series	PEX + prednisone 10 mg/day	18	LA and/or IgM/IgG aCL	FD (39), PE/IUGR (9)	none	18 (100)	6 (33.3)	none	LoE 4
Bramham et al. (2011)	pros	Prednisolone 10 mg/day from positive test to 14th WG	23	LA (13), IgM aCL (10), and IgG aCL (8)	REM (16), FD (1), and REM/FD (1)	thrombosis (6)	14(60.9)	6 (42.8)	none	LoE 2b
De Carolis et al. (2015)	case report	HCQ 400 mg	1	triple aPL	FD	none	1 (100)	1 (100)	none	LoE 4
Mekinian et al. (2015)	retro	HCQ 200–400 mg	14	n.r	REM/FD (9)	thrombosis + pregnancy morbidity (5)	11/14 (78)	n.r	none	LoE 2b
Mayer-Pickel et al. (2017)	case report	IA	1	n.r	REM (4)	none	1 (100)	0 (0)	none	LoE 4
Mekinian et al. (2017)	retro	HCQ 200 ± GCs	49	LA (36), IgM aCL (20), IgG aCL (57), IgM aβ2GPI (12), IgG aβ2GPI (22), triple aPL (22)	REM (15), FD (23), PB (11), PE/HELLP (5), and CAPS (3)	venous thrombosis (8) and arterial thrombosis (6)	43 (86)	29 (59.2)	n.r	LoE 2b
Ruffatti et al. (2018)	retro	HCQ 200–400 mg	20	triple aPL or LA and/or aCL or anti- aβ2GPI	FD (40)	none	19 (95)	1 (5)	1 (1.1) dermatitis	LoE 2b
		LDS 10–20 mg/day	9				5 (55.5)	2 (22.2)	none	
		HCQ 200–400 mg + LDS 10–20 mg/day	9				5 (55.5)	5 (55.5)	none	
		IVIG 2 gr/kg/month	2				2 (100)	2 (100)	none	
Alijotas-Reig et al. (2019)	pros	anti–TNF-α	12	LA (6), IgM/IgG aCL (9), and IgM/IgG aβ2GPI (5)	REM (10) and FD (10)	none	6/12(50%)	n.r	none	LoE 2b
Urban et al. (2020)	case-series	IVIG 0.8 gr/kg/monthly + LDS 5–7.5 mg/day + HCQ 200 mg/day	4	triple aPL (2) and LA + IgM aCL (1)	REM/FD (2) and PE + FD (1)	thrombosis (1)	5/5 (100%) ^	3 (75)	none	LoE 4

LA, lupus anticoagulant; aCL, anti-cardiolipin antibodies; anti-^®^2 GPI, anti-^®^2 glycoprotein I; IgG, immunoglobulin G; IgM, immunoglobulin M; REM, recurrent early miscarriage; FD, fetal death; PE, preeclampsia; PB, premature birth; HELLP, hemolysis, elevated liver enzymes, and low platelet; IUGR, intrauterine growth restriction; IA, immunoadsorption; PEX, plasma-exchange; TNF-α, tumor necrosis factor alpha; IVIG, intravenous immunoglobulin; LDS, low-dose steroids; HCQ, hydroxychloroquine; WG, week of gestations.

nr. not reported.

^twin pregnancy.

The first description of additional treatment in conjunction with SoC used to prevent pregnancy failure in refractory APS pregnancies dates back to 2007 (El Haieg et al., 2007). Eighteen pregnancies were treated, from 7.08 ± 0.6 SD weeks of gestation, with prednisone 10 mg/daily + PEX for three sessions/week until the LAC activity was suppressed and IgG aCL lowered. They reported a live birth rate of 100%. The following pregnancy complications were observed: mild pre-eclampsia in 5.5%, preterm deliveries in 22.2%, IUGR in 11.1%, and oligoidramnios and fetal distress in 16.6%. Four years later a small prospective observational study evaluated the efficacy of prednisolone 10 mg daily (Bramham et al., 2011), from the time of positive pregnancy test to 14 weeks of gestation, achieving 14/23 (60.9%) of live births. The authors state that seven (64%) of 11 women with previously fetal losses < 10 weeks of gestation had live births, whereas two (29%) of seven women with fetal losses >10 weeks of gestation had live birth when treated with prednisolone 10 mg daily. Six (42.8%) severe pregnancy complications were registered.

Hydroxychloroquine alone or in association with LDS has been employed as an additional treatment to prevent pregnancy losses in refractory APS pregnancies. A retrospective European multicenter study (Mekinian et al., 2015) reported 11/14 (78%) live births, while HCQ 200–400 mg was added to the SoC. It is worth noting that all women experienced pregnancy losses while under treatment with LDA + LMWH in 11 cases and LMWH in three cases. These data were confirmed by two further multicentric retrospective studies (Mekinian et al., 2017; Ruffatti et al., 2018). The former (Mekinian et al., 2017), by comparing the pregnancy outcomes of 49 women with their previous pregnancies while treated with SoC, reported a significantly increased live birth rate from 13 to 86%. At least one severe pregnancy complication was registered in 29/49 (59.2%) subsequent pregnancies. Seven (15%) of them were treated with HCQ ± LDS in their previous pregnancies. The latter (Ruffatti et al., 2018) showed a live birth of 95% in pregnancies treated with HCQ 200–400 mg alone and 55.5% in those treated with HCQ 200–400 mg + LDS in addition to the SoC. Severe pregnancy complications were registered in 5 and 55.5% of those treated with HCQ 200–400 mg alone and HCQ 200–400 mg + LDS in addition to the SoC, respectively.

Interestingly, results are reported by using IVIG ≤2 gr/kg/monthly alone or in association with HCQ and LDS, albeit the low number of patients treated. Two case series (Ruffatti et al., 2018; Urban et al., 2020), treating respectively two and four pregnancies, achieved both a 100% of live birth rate. However, a higher number of severe pregnancy complications were observed, respectively, in 100% and 75% of the pregnancies. It is worth noting that none of the women had a live birth while being treated with SoC, even when the latter was associated with HCQ, as reported in four pregnancies by Urban et al. (2020).

One promising treatment is TNF-α inhibitors. In a pilot study (Alijotas-Reig et al., 2019), twelve women underwent *in vitro* insemination to maximize the embryonic quality and ensure the best timing for initiation of TNF-α inhibitor therapy. The authors observed 6/12 (50%) live births, 3/12 (25%) miscarriages, and 3/12 (25%) embryo implantation failure. No pregnancy complication has been reported.

Overall, among the 162 (37.3%) refractory APS pregnancies, 35 (21.6%) were treated with HCQ 200–400 mg, 58 (35.8%) with HCQ 200–400 mg + LDS 10–20 mg, 32 (19.8) with LDS 10–20 alone, 6 (3.7%) with IVIG <2 gr/kg/monthly ± HCQ 200–400 mg ± LDS 5–7.5 mg, 19 (11.7%) with PEX/IA weekly ± LDS 10–20 mg, and 12 (7.4%) with anti-TNFα (certolizumab or adalimumab).

Altogether, by using additional treatments, a live birth rate of 130 (80.2%) was achieved. Treatment regimen employing IVIG <2 gr/kg/monthly ± HCQ ± LDS, as well as PEX/IA ± LDS, led to the higher live birth rates, 100% for both. Moreover, HCQ 200–400 mg alone showed a higher live birth rate than HCQ + LDS (88.6% *vs*. 82.7%). Instead, LDS alone and TNF-α inhibitors registered lower live birth rates of 59.4% and 59%, respectively. Furthermore, 55 (48.6%) pregnancy complications were registered. A treatment regimen with HCQ 200–400 mg alone showed the lower pregnancy complications 16.7%, while HCQ + LDS and IVIG <2 gr/kg/monthly ± HCQ ± LDS showed the higher ones of 70.8 and 83.3%, respectively. One (0.6%) case of dermatitis during treatment with HCQ was observed.

### Additional Treatment to Prevent Pregnancy Failure in Refractory/High-Risk APS Pregnancies

The studies investigating the effects of additional treatments in the high-risk/refractory APS pregnancies are reported in Table 2.

**TABLE 2:** Studies investigating the effects of additional therapy in high-risk/refractory pregnancies in antiphospholipid syndrome.

Author, year	Study design	Additional treatment protocol	Number of pregnancies	aPL profile	Pregnancy morbidity, (n)	Thrombosis, (n)	Live birth, n (%)	Pregnancy complications, n (%)	Adverse events, n (%)	Level of evidence
Ruffatti et al. (2007)	case series	PEX weekly from first/second trimester	4	triple aPL	FD (1), REM (1)	thrombotic microangiopathy (2), venous thrombosis (1), and arterial thrombosis (1)	2 (50)	2 (50)	none	LoE 4
		PEX + IVIG 2 gr/kg/monthly from 21st to 29th WG	2	triple aPL	PE (2)	venous thrombosis (1) and thrombotic microangiopathy (1)	2 (100)	2 (100)	none	
Stojanovic et al. (2007)	case report	IVIG 5 g/month + DEXA1.5–2.5 mg/day from 13th to36th WG	1	triple aPL	PE	none	1 (100)	0 (0)	none	LoE 4
Watanabe et al. (2014)	case series	IVIG 0.4 g/kg 5 day/monthly + prednisolone 10–20 mg/day from the detection of FHB	4	triple aPL	PE (1) and FD (2)	arterial thrombosis (1) and venous thrombosis (1)	4 (100)	1 (33.3)	none	LoE 4
Lefkou et al. (2014)	case report	pravastatin 20 mg/d from 23rd WG	1	LA	PE	venous thrombosis	1 (100)	1 (100) IUGR	none	LoE 4
Ruffatti et al. (2014)	Retro	PEX + IVIG 2 gr/kg/monthly	7	triple aPL (14)	PE/HELLP/IUGR (13)	thrombosis (13)	6 (85.7)	0 (0)	none	LoE 2b
		IVIG 2 gr/kg/monthly	5				3 (60)	0 (0)	none	
		IVIG 2 gr/monthly + LDS 10–20 mg of prednisone	3				3 (100)	1 (33.3)	none	
		PEX weekly	4				4 (100)	0 (0)	none	
		IVIG 2 gr/kg/monthly + IA	2				2 (100)	0 (0)	none	
Mayer-Pickel et al. (2015)	case report	PEX weekly from 19th WG	1	LA	none	venous thrombosis	1 (100)	1 (100)	none	LoE 2b
Ruffatti et al. (2015)	retro	IVIG 2 gr/kg/monthly + IA	4	triple aPL	PE (2) and FD (2)	thrombosis (4)	4 (100)	2 (50)	none	LoE 2b
		PEX + IVIG 2 gr/kg/monthly	14		PE (9), FD (11)	thrombosis (9)	13 (92.8)	5 (35.7)	none	
Ruffatti et al. (2016)	Pros	PEX + IVIG 2 gr/kg/monthly	18	triple aPL	HELLP (4), PE (6), and IUGR (1)	thrombotic microangiopathy (4), venous thrombosis (4), and arterial thrombosis (4)	17 (94.4)	4 (26.7)	none	LoE 2b
Lefkou et al. (2016)	case-control	pravastatin 20 mg/d	11	LA (7), IgM aCL (3), IgM aβ2GPI (1), aCL (1)	FD (4), HELLP (2), PE (2), stillbirth (3), and placental abruption (1)	venous thrombosis (1)	11 (100)	1 (9.1)	none	LoE 2b
Mayer-Pickel et al. (2017)	case series	PEX weekly from 18 to 25 WG	3	n.r	PE (2)	thrombosis (3)	3 (100)	1 (33.3)	none	LoE 4
Ruffatti et al. (2018)	Retro	HCQ 200–400 mg	74	triple aPL or LA and/or aCL or aβ2GPI	PE/HELLP/IUGR (68)	thrombosis (100)	63 (85.1)	19 (25.7)	none	LoE 2b
		LDS 10–20 mg/day	27				22 (81.5)	14 (51.8)	none	
		HCQ 200–400 mg + LDS 10–20 mg/day	10				7 (70)	4 (40)	none	
		IVIG 2 gr/kg/monthly	14				13 (92.8)	4 (28.6)	none	
		PEX weekly	8				7 (87.5)	5 (62.5)	none	
		PEX + IVIG 2 gr/kg/monthly	21				20 (95.2)	3 (14.3)	none	
Rovere-Querini et al. (2018)	case report	ECU 600 mg + HCQ 300 mg	1	triple aPL	REM	venous thrombosis	1 (100)	1 (100)	none	LoE 4
Colpo et al. (2020)	Retro	PEX + IVIG 2 gr/kg/monthly	26	triple aPL	PE/HELLP/IUGR (17)	thrombosis (11)	23 (88.5)	9 (34.6)	none	LoE 2b
Lefkou et al. (2020)	case-control	pravastatin 20 mg/d	7	triple aPL	REM (7), PE (3), and PB (2)	nr	7 (100)	nr	none	LoE 2b

aPL, antiphospholipid syndrome; LA, lupus anticoagulant; aCL, anti-cardiolipin antibodies; anti-^®^2 GPI, anti-^®^2 glycoprotein I; IgG, immunoglobulin G; IgM, immunoglobulin M; REM, recurrent early miscarriage; FD, fetal death; PE, preeclampsia; PB, premature birth; HELLP, hemolysis, elevated liver enzymes, and low platelets; IUGR, intrauterine growth restriction; PEX, plasma exchange, TNF-α, tumor necrosis factor alpha; IVIG, intravenous immunoglobulin; LDS, low-dose steroids; HCQ, hydroxychloroquine; ECU, eculizumab. nr. not reported.

The first connotation of high-risk pregnancies in antiphospholipid antibody syndrome and consequently their management dates back to the early 2000s. A case series (Ruffatti et al., 2007) described four pregnancies treated with PEX weekly from the first trimester and two with PEX weekly + IVIG 2 gr/kg/monthly from the second trimester (when signs of pregnancy complications were detected) reporting 2 (50%) and 2 (100%) of live births, respectively. As many severe pregnancy complications have been observed. It is worth noting that none of these women ended a successful pregnancy while treated with the SoC. Subsequently, a European multicenter study (Ruffatti et al., 2014) demonstrated in seven patients treated with PEX + IVIG 2 gr/monthly and five receiving IVIG 2 gr/monthly alone live birth rates of 85.7% and 60%, respectively. Moreover, four patients treated with PEX alone, three with IVIG 2 gr/monthly + LDS, and two with IVIG 2 gr/monthly + immunoadsorption all had a live birth rate of 100%. One (33.3%) severe pregnancy complication was reported in the patients treated with IVIG 2 gr/monthly + LDS. More consistent results were derived from the publication of a prospective study (Ruffatti et al., 2016). Eighteen pregnancies of 14 women underwent treatment with PEX or immunoadsorption weekly plus fortnightly IVIG 1 gr/kg, achieving a live birth rate of 94.4%, and 11.1% of severe pregnancy complications. The live birth rate was significantly higher than that following the patients’ previous pregnancy while on treatment with SoC. Also, severe pregnancy complications resulted significantly lower with respect to their previous pregnancies. These findings have been confirmed by other cohort studies and case series (Table 2). Also, a case report (Stojanovic et al., 2007), a case series (Watanabe et al., 2014), and a multicenter retrospective study (Ruffatti et al., 2018) highlighted the benefit of IVIG therapy in high-risk primary APS pregnancies.

In 2018, a European multicenter retrospective study (Ruffatti et al., 2018), reporting the results of HCQ 200–400 mg alone or associated with LDS additional therapy protocol in 74 and 27 pregnancies, respectively, was published. The pregnancies treated with HCQ 200–400 mg alone with respect to the counterparty treated with HCQ 200–400 mg with LDS registered a higher live birth rate (85.1% *versus* 70%) and a lower frequency of severe pregnancy 25.7% *versus* 40%). This study highlighted the importance of the dosage and the timing of HCQ as the high (400 mg) versus low (200 mg) doses of HCQ and its administration before versus during pregnancy were associated with a significantly higher live birth rate. Moreover, HCQ appeared particularly efficacious in the primary APS patients with no history of thrombosis. Interesting data come from the use of pravastatin in pre-eclamptic patients with APS. A first case report (Lefkou et al., 2014) followed by a pilot case-control study (Lefkou et al., 2016) of 21 pregnancies was published. In the case-control study, eleven patients received pravastatin (20 mg daily) in addition to LMWH/LDA at the onset of pre-eclampsia and/or IUGR, while the control group of ten patients received only LMWH/LDA. All pregnancies treated with both pravastatin and LMWH/LDA ended with a viable infant. Moreover, they exhibited increased placental blood flow and improvements in pre-eclampsia features. These beneficial effects were observed as early as 10 days after pravastatin treatment onset. In the control group, all deliveries occurred preterm and only six of 11 neonates survived. Furthermore, in a subsequent study, Lefkou et al. (2020), in addition to confirming the results of the previous study, hypothesized that triple therapy with pravastatin + LMWH + LDA improves placental hemodynamics and thus the outcome of pregnancy through a nitric oxide–dependent mechanism.

Among the 272 (62.7%) high-risk/refractory APS pregnancies, 74 (27.2%) were treated with HCQ 200–400 mg, 10 (3.7%) with HCQ 200–400 mg + LDS 10–20 mg, 30 (11%) with LDS 10–20 mg alone, 19 (6.9%) with pravastatin 20 mg, 1 (0.4%) with eculizumab 600 mg + HCQ 300 mg, 20 (7.4%) with PEX weekly, 88 (32.4%) with PEX weekly + IVIG 2 gr/kg/monthly, 24 (8.8) with IVIG 2 gr/kg/monthly, and 6 (2.2) with IA weekly + IVIG 2 gr/kg/monthly. Following additional treatment protocols, a live birth rate of 240 (88.2%) was obtained. Moreover, 66 (27.6%) pregnancy complications were reported. The higher live birth rate was achieved, following treatment with pravastatin 20 mg (100%), eculizumab 600 mg + HCQ 300 mg (100%), IA weekly + IVIG 2 gr/monthly (100%), and PEX weekly + IVIg 2 gr/kg/monthly (92%) and the lower one with HCQ 200–400 mg + LDS 10–20 mg (70%). On the other hand, the lowest frequency of severe pregnancy outcomes was reported in pregnancies treated with PEX weekly + IVIg 2 gr/kg/monthly (11.1%). No adverse event was registered.

## Discussion

In this systematic review of the literature, we aimed to summarize the currently available literature on the efficacy and safety of additional treatment protocols used in conjunction with SoC, according to risk stratification in APS pregnancies.

Ruffatti et al. (2014) showed that additional treatment gives a higher live birth rate, but which treatment and when to use it was not possible due to the low number of the cohort. Twenty-one studies were eligible for a total of 434 pregnancies. Of these, 37.3% were refractory, and 62.7% were high-risk/refractory obstetric APS. Following the additional treatment protocols, high live birth rates (80.2 % and 88.2%, respectively) and low severe pregnancy complication rates (48.6 % and 27.6%, respectively) were observed both in refractory and high-risk/refractory APS pregnancies.

In the refractory APS pregnancies, the most frequent additional therapy protocols were HCQ 200–400 mg ± LDS 10–20 mg and HCQ 200–400 mg alone, in 35.8% and 21.6% of the cases, respectively. In the refractory APS, HCQ 200–400 mg showed a higher live birth rate with respect to LDS 10–20 mg and anti-TNFα, while there was almost similar live birth rates with respect to HCQ 200–400 mg ± LDS 10–20 mg, IVIG <2 gr/kg/monthly ± HCQ 200–400 mg ± LDS 5–7.5 mg, or PEX weekly. Furthermore, HCQ 200–400 mg showed a lower severe pregnancy complication rate than LDS, HCQ ± LDS, and IVIG.

HCQ, an antimalarial drug, is used widely in autoimmune rheumatic diseases. Recently, *in vitro* studies (Albert et al., 2014; Marchetti et al., 2014) have demonstrated that HCQ reverses the aPL inhibition of trophoblast interleukin-6 secretion and partially limits aPL inhibition of cell migration, as well as restores trophoblast differentiation affected by anti-β2GP1 antibodies. Both these pathogenetic mechanisms are involved in the inhibition of syncytialization, an essential process for the replenishment of the syncytiotrophoblast, leading to an inadequate invasion of the maternal spiral arteries, a reason for early miscarriages. Thus, these studies suggest a role for HCQ to prevent recurrent pregnancy loss refractory to the SoC. Moreover, *in vivo* studies (Mekinian et al., 2015; Mekinian et al., 2017; Ruffatti et al., 2018) point out the efficacy of HCQ in preventing pregnancy loss in refractory APS. These studies had interesting findings to help clinicians when treating these women. First, HCQ at a higher dose (HCQ 400 mg) was observed to be more efficacious than a lower dose (HCQ 200 mg) (Mekinian et al., 2015). Second, HCQ initiated during conception was associated with an increase in the live birth rate than one started during pregnancy (Ruffatti et al., 2018). Third, HCQ was less effective in patients with thrombosis than in those without (Ruffatti et al., 2018) in preventing pregnancy loss. To date, one case of adverse events has been observed. Currently, there are three ongoing multicenter randomized control trials of HCQ 400 mg *versus* placebo, the HYPATIA (Schreiber et al., 2017), the HIBISCUS (Belizna et al., 2018), and the BBQ study (Pasquier et al., 2019), respectively, which explores the efficacy of HCQ in refractory APS pregnancies, informing clinicians about its efficacy and safety.

In high-risk/refractory obstetric APS the most frequent additional therapy protocols were PEX weekly + IVIG 2 gr/kg/monthly and HCQ 200–400 mg alone, respectively 32.3 % and 27.2%.

The live birth rate as a result of the various additional therapeutic protocols varied between 70% and 100%. To note, treatments such as pravastatin, eculizumab 600 mg + HCQ 300 mg, and IA weekly + IVIG 2 gr/kg/monthly resulted in 100% viable infants and PEX weekly + IVIG 2 gr/kg/monthly resulted in 92% viable infants. On the other hand, PEX weekly + IVIG 2 gr/kg/monthly conferred a lower rate of severe complications than PEX weekly, HCQ 200–400 mg and LDS 10–20 mg alone, and the HCQ 200–400 mg ± LDS 10–20 mg, Treatment with pravastatin, eculizumab 600 mg + HCQ 300 mg, and IA weekly + IVIG 2 gr/kg/monthly constitute only 9.6% of the high-risk/refractory pregnancies studied. Taken together, PEX weekly + IVIG 2 gr/kg/monthly is the additional treatment associated with the higher live birth rate and the lower rate of severe pregnancy complications. The high-risk/refractory pregnancies, as shown in Table 2, have had for the most part severe pregnancy complications such as PE, HELLP, and IUGR or late fetal loss. Recently, studies on the etiopathogenetic role of aPL on the placenta (De Simone et al., 2006; Chen et al., 2009; Marchetti et al., 2013; Tong et al., 2015) observed an impaired trophoblast invasion mediated by the aPL-altering expression of adhesion molecules such as placental growth factor, vascular endothelial growth factor, and soluble FmS-like kinase I, as well as complement and coagulation cascade activation, which are thought to be responsible for late pregnancy loss and preeclampsia. PEX is an extracorporeal blood purification technique, demonstrated safe in treating different autoimmune diseases during pregnancies (Colpo et al., 2019). As described back in 1914 by Abel et al. (1914), the principle on which plasmapheresis is based is the “take away”, pathogenetic autoantibodies. Probably by reducing autoantibody levels, PEX helps to eliminate the pathogenic noxa which alters the transformation of the spiral arteries and stimulates the activation of the complement system and the coagulation cascade. IA is based on the same principles but is more selective, removing only the IgG aPL autoantibodies, and thus might be reserved for patients with only IgG aCL and/or anti-β2GPI. Also, IVIG, a blood product employed in numerous autoimmune diseases, contributes to reducing autoantibody levels through the presence of anti-idiotype antibodies that bind aPL and/or inactivation of B clones leading to reduced autoantibody production (Sherer et al., 2000; Tenti et al., 2015). The studies focused on the efficacy and safety of therapeutic apheresis procedure in combination with IVIG (Ruffatti et al., 2007; Ruffatti et al., 2016; Ruffatti et al., 2018; Colpo et al., 2019; Hoxha et al., 2020) found out that initiating the treatment protocol at the first signs of placental insufficiency led to viable infants. However, despite the high live birth rate, a higher number of severe pregnancy complications and therefore fetal/neonatal complications were encountered than the counterpart starting treatment from early pregnancy.

So it is crucial to identify risk factors and start additional treatment protocols from the beginning of the pregnancy.

Promising additional treatment protocols to prevent recurrent pregnancy loss, as opposed to high-risk/refractory conditions, are anti-TNFα and pravastatin. Also, there is an ongoing clinical trial, the IMPACT study (NCT03152058), which is evaluating certolizumab therapy to improve pregnancy APS in refractory and/or high-risk pregnancies. Certolizumab, an Fc-free anti-TNF drug, showed a lack of placental transfer during pregnancy in women with chronic inflammatory diseases (Mariette X., et al., 2018). If the preliminary data are confirmed by this clinical trial, in the near future, certolizumab might be considered to prevent recurrent losses in refractory APS pregnancies. Preclinical and clinical studies on pravastatin show very interesting results. In fact, starting the therapy at the onset of preeclampsia or IUGR led to a rapid improvement of the hemodynamic parameters of the uterine arteries, resulting in higher live birth rates and lower pregnancy complications. The fewer high-risk/refractory pregnancies (6.9%) are treated with pravastatin and safety concerns as it may be teratogenic and are still classified as category X by the States Food and Drug Administration, which restricts its use. If future large-scale studies confirm its efficacy and above all reassure its safety, it could be a valid additional therapy protocol for high-risk/refractory obstetric APS.

There are several limitations to this study. First, the retrospective design of most of the studies and the presence of case series and case reports constitutes in itself a limitation to the study. Second, the studies considered are not homogeneous, both for the dosage of drugs used and for the timing of the introduction of the therapy. The strengths of the study are the large number of pregnancies analyzed and the homogeneity of the study population, consisting exclusively of primary antiphospholipid syndrome (APS) refractory or high-risk/refractory to the SoC.

In conclusion, this systematic review of the literature summarizes the current evidence of additional therapy protocols used in preventing recurrent losses in the APS pregnancies who fail the SoC. Using these protocols, a high number of live births and a low number of pregnancy complications were achieved. Overall, HCQ 200–400 mg could be considered an additional therapy protocol option in preventing recurrent losses in refractory APS. On the other hand, PEX weekly + IVIG 2 gr/kg/monthly could be taken into consideration in the management of high-risk/refractory APS. The results presented provide clinicians with up-to-date knowledge in the management of APS pregnancies according to risk stratification.

## Data Availability

The raw data supporting the conclusion of this article will be made available by the authors, without undue reservation.
