# Supplementary material for: Treatment of Refractory/High-Risk Pregnancies With Antiphospholipid Syndrome: A Systematic Review of the Literature
Source: Front Pharmacol. 2022 May 19;13:849692. doi: 10.3389/fphar.2022.849692 (PMC9160870; doi:10.3389/fphar.2022.849692)
Supplement: Supplementary file 2 [file DataSheet1.docx]

**APPENDIX I**

**Topic: “**How to treat pregnant women with refractory or high-risk/refractory obstetric antiphospholipid syndrome?”

**Methods**

**Database/s to search:** Pubmed, Web of Science, Cochrane database and www.clinicaltrials.gov

**Keywords:** obstetric antiphospholipid syndrome, antiphospholipid syndrome, pregnancy, additional treatments, refractory antiphospholipid syndrome, hydroxychloroquine, low-dose steroids, intravenous immunoglobulins, plasma exchange, rituximab, eculizumab, certolizumab, adalimumab, etanercept, statins, adverse events.

**Search strategy:**

refractory antiphospholipid syndrome and pregnancy

antiphospholipid syndrome and pregnancy and additional treatments

antiphospholipid syndrome and pregnancy and hydroxychloroquine

antiphospholipid syndrome and pregnancy and low-dose steroids

antiphospholipid syndrome and pregnancy and intravenous immunoglobulins

antiphospholipid syndrome and pregnancy and plasma exchange

antiphospholipid syndrome and pregnancy and rituximab

antiphospholipid syndrome and pregnancy and eculizumab

antiphospholipid syndrome and pregnancy and certolizumab/adalimumab/etanercept

antiphospholipid syndrome and pregnancy and statins

Number of selected studies were: n. 21

**Type of studies/publications to include in the search:**

- Randomized controlled trials (published) n. 0
- Observational studies Cohort studies n. 9

Case-Control studies n. 2

Case Series n. 5

Case report n. 5

Papers reporting insufficient data were excluded.

**Limits applied to research**

Gender: female

Type of patients: primary obstetric antiphospholipid syndrome only

Timing: February 2006-October 2021

Language: English

**Study identification and data extraction**

Full-text articles were screened and selected analyzing titles and abstracts. After the screening phase, were evaluated the selected abstracts and the full-text of these studies to determine eligibility. Data extraction was also performed. After that, the results of the analysis of literature were summarized.

**Results**

**PICO specific question**: which is the most effective and safe additional treatment to conventional therapy for inducing a favorable pregnancy outcome in women with refractory or high risk obstetric APS?

**PICO’s Strategy**

- **P**– Population = women with refractory or high risk primary obstetric APS
- **I**– Intervention = additional treatments to conventional therapy
- **C**– Comparison = with a control group on placebo or on different therapy
- **O**– Outcome = outcome of pregnancy (live birth, pregnancy loss, maternal complications, fetal complications, side effects)

**Intervention considered for the systematic review of the literature**

Hydroxychloroquine

Low-dose steroids

Intravenous immunoglobulins

Plasma exchange

Rituximab

Eculizumab

Certolizumab/Adalimumab/Etanercept

Statins

Combined treatments
